# Crossing Countries and Crossing Ages: The Difficult Transition to Adulthood of Unaccompanied Migrant Care Leavers

**DOI:** 10.3390/ijerph18136935

**Published:** 2021-06-28

**Authors:** Federica Gullo, Laura García-Alba, Amaia Bravo, Jorge F. del Valle

**Affiliations:** Department of Psychology, University of Oviedo, 33003 Oviedo, Spain; garciaalblaura@uniovi.es (L.G.-A.); amaiabravo@uniovi.es (A.B.); jvalle@uniovi.es (J.F.d.V.)

**Keywords:** unaccompanied migrant young people, transition to adulthood, leaving care, child welfare, aftercare support, migration, special migrants’ populations

## Abstract

The social changes experienced in many countries have prolonged the transition to adult life for young people. That being said, those who leave child care cannot afford this privilege, in that they do not benefit from the same support and resources, having to confront an accelerated transition which exposes them to increased risk of negative outcomes and social exclusion. Moreover, this transition might be even riskier for unaccompanied migrant care leavers, who are four times as vulnerable, given their status as young people in care, as adolescents, as migrants and being unaccompanied. This paper seeks to explore the profiles, needs, and experiences of unaccompanied young migrants in comparison with other care leavers. Data were collected by means of a semi-structured interview to explore their pre-care, in-care, and aftercare experiences. A highly specific profile of unaccompanied young migrants has been revealed that differs from the other care leavers in terms of worse educational, occupational, and economic outcomes, limited support networks, and more obstacles to accessing aftercare supports. Conversely, they also exhibited some strengths, such as having less pre-care, in care, and aftercare traumatic experiences, less psychological distress and fewer risky behaviors compared with other care leavers.

## 1. Introduction

During the past century, economic and social changes have brought about a global delay in young people’s process of emancipation, making their entry into adult roles more gradual and non-linear [1]. Arnett [2] described the late teens and early twenties as a developmental period of emerging adulthood, characterized by changes and explorations in education, work, and love, and restricted to cultures of highly industrialized societies that postpone the acquisition of responsibilities until the late twenties. Nevertheless, he recognized that the increasing globalization of the world economy opened the possibility that emerging adulthood could become a normative period for young people worldwide, allowing the prolongation of exploration and freedom even in developing countries [2]. Indeed, within the last century, economic, social and cultural globalization has transformed the experiences and conceptions of transition to adulthood also among young people from non-Western cultures [3]. Mitchell [4] referred to the period during the twenties and into the thirties as the boomerang age, an anteroom of full adulthood in which people alternated periods of leaving and returning to the family home. In Europe, it has been estimated that around 50% of young people aged 18 to 34 live with their parents and 29.5 has been calculated to be the average age at which Spanish youth “fly the nest” (EUROSTAT, 2019). This emancipation process is even more challenging for young people who have lived under child care intervention until the majority of age, a time when guardianship concludes and they are suddenly forced to embark on a path towards independence. Transition to adulthood has been traditionally defined as the assumption of new roles and tasks related to the acquisition of autonomy and social integration, that culminates in the achievement of education, training, work, mature relationships, financial and housing independence [5]. Care leavers find themselves having to face this process many years earlier than their non-care experienced peers in Spain. The accelerated and compressed transition to adult life, with fewer resources and support, exposes them to high risk of social exclusion [6] and poorer outcomes, in terms of limited education, unemployment or worse working conditions, housing instability, poverty, mental health issues, substance abuse, problems with the law, early parenting, limited social support, and dependence on social assistance [5,7,8,9,10]. In fact, care leavers have been identified as one of the most vulnerable and disadvantaged groups in society [11]. This situation can be even worse for specific subpopulations of this group, such as unaccompanied young migrants who arrived in a foreign country as minors, without the protection of a family member or an adult responsible for them (Council of Europe, 1977). In Spain, the term used to refer to this group is “unaccompanied foreign minors” instead of “unaccompanied asylum-seeking children”, since they have not needed to seek asylum to be protected. Their guardianship is assumed by the regional authority, which, in accordance with national law, has the same obligation to protect them as if they were native minor. Consequently, we will use the term “unaccompanied young migrants” (UYM) to refer to this group of care leavers.

The arrival of these young people has increased progressively in the last two decades in many countries and Spain has typically been one of the main gateways to Europe for them. Those who arrive do not usually flee from countries in conflict where their security is in danger, but rather from countries with a worse socioeconomic situation, mostly Morocco [12]. They arrive with an economic migratory objective, wanting to get a job and achieve a better life [12], also influenced by the European myth, and the consequent belief of being able to achieve their dreams quickly and successfully [13]. The magnitude of this phenomenon cannot be precisely quantified, due to the different methods and criteria used to collect data, inconsistencies in the data provided by different sources, and the fact that some young migrants have yet to enter into childcare, but have remained on the streets or have been recruited by criminal and mafia networks [14,15]. In Spain, the migratory phenomenon has grown since the late 1990s, directly impacting the child services and putting enormous pressure on existing resources [12]. Residential care has been the most frequently used intervention (99% in 2019) for UYM in our country [16]. According to the latest data, in 2019 there were 11.380 UYMs referred to residential child care, which represent 49% of young people in this out-of-home measure, with a 19% increase compared to 2018 [16]. Moreover, addressing their special needs has been challenging for child care services, as they are typically adolescents close to majority age, requiring swift preparation for transition to adulthood, if they are to be socially and occupationally integrated [17]. Such transitions have been especially complicated for unaccompanied migrant care leavers who have found themselves in a foreign country whose language and customs they do not know [17]. They enter this process with even scarcer resources than other care leavers, in terms of home, job, training, money, support [18], often with no protection and in an irregular situation overnight [19], carrying out the care-leaving process in a transnational space [20] in which both “there and here” have relevance [21]. Their particular condition entails increased vulnerability, inasmuch as they suffered four elements of vulnerability: they were in care, adolescents, unaccompanied, and migrants and they found themselves in an ambiguous legal situation, since their stay in the country depends on different laws. They should enjoy the rights recognized by the United Nations Convention on the Rights of the Child (UNCRC, 1991), the Spanish constitution, and the national laws for the protection of minors, but as migrants, they are also subject to immigration law, which implies constant instability and uncertainty. Until legal age, their condition as being underaged prevails. However, when they reach the majority age, they can lose the protection of the authorities and be considered adult migrants, with all the incumbent consequences if they have not managed to legalize their immigrant status before then [13]. In this case, the transition into adulthood is accompanied by the transition into illegality [22]. To aggravate the situation further, local authorities answer very differently to the needs of this vulnerable group, affecting their preparation for independent life [23] and future integration into the host society [24], and the scarcity of leaving care support services complicates their possibilities of accessing such services, leaving them alone in this process [25].

In the last several decades, there has been growing interest in international research dedicated to care leavers, but the transition into adulthood of a subgroup as unique as UYM has remained largely unexplored, particularly at a national level. Some studies have focused on specific aspects of UYMs’ adaptation to their new life situation, such as their mental health or well-being [13], educational level [26] or employability [27], but there has been a paucity of holistic approaches. Furthermore, studies regarding UYMs in child care have increased [28], but studies that examine their transition to adulthood are rare [23].

Therefore, the first purpose of this paper was to study the characteristics of UYM care leavers with respect to several aspects having to do with their pre-care and in-care experiences. The second purpose was to explore their after-care situations and needs in relevant areas of social integration, such as education and training, jobs, accommodations, income, health, and support networks. Finally, in every analysis carried out, the differences between UYMs and the rest of care leavers were taken into account, with the hypothesis that outcomes would be worse for the former in many of the areas assessed. Findings will provide evidence about the specific characteristics of UYMs compared to their care-experienced peers without a migration background, highlighting their different profiles, strengths and weaknesses.

## 2. Materials and Methods

### 2.1. Sample

Participants were care leavers from different Spanish regions (Catalonia, Basque Country, Cantabria, Madrid, Castile-Leon, Castile-La Mancha, and Galicia) who remained connected to aftercare services for transition into adulthood. The sample was composed of 141 males aged 18–25 (*M* = 19.17, *SD* = 1.45) and divided into two groups: 68 unaccompanied young migrant (UYM) and a comparison group (CG) consisting of other care leavers from Spanish families or with a family history of immigration (*n* = 73). Most of the UYMs were 18 (54%) or 19 (29%) years old, with only 16% aged 20 years or older, while in the CG, they were better distributed among the different ages (30%, 32%, and 38%, respectively). Nevertheless, the UYMs average age (18.97, *SD* = 1.61) was not significantly different from that of the CG (19.36, *SD* = 1.26; [*t* (139) = 1.59, *p* = 0.115]). The reason for having only male participants was due to the lack of female UYMs in care. According to the latest data, they represent a mere 6.8% of UYMs in our child care services [14], making it difficult to find female UYM participants.

The UYMs were mostly from North Africa (72%) (notably Morocco and a few from Algeria), Sub-Saharan Africa (25%) (from countries like Senegal, Gambia, Guinea, and Nigeria), and 3% from Asia. In contrast, the young people from immigrant families in the CG group were mostly from Latin America (57%) or Africa (38%, especially Sub-Saharan), and 5% from Eastern Europe.

### 2.2. Instruments

Data collection was performed using a qualitative, semi-structured interview created specifically for this research to gather relevant information about the participants’ profile and their current and past situations. In addition to sociodemographic characteristics, such as age and country of origin, the following areas were explored: (a) previous experiences in child care, including time spent in care, placement changes, and victimization; (b) health and risk behaviors, such as health problems, intellectual disability, mental health treatment, substance use, suicidal behavior, delinquency, and unexpected pregnancy by a partner; (c) current education, work, economic, and accommodation status; (d) social support network, especially from family, friends, partners, and reference adults, and (e) aftercare services received in different areas.

### 2.3. Procedure

In order to have a global vision of the services available in the national territory, data were collected in different regions which have been chosen for having transition services that work with a considerable number of young people and that are among the best developed and with the longest experience in the country. Prior authorization for the study was obtained from the child care authorities in each region. Then, the respective aftercare support agencies were informed about the study objectives and methods. A convenience sampling method was used to select participants; the teams that work with care leavers in each Autonomous Community contacted the participants to propose that they participate in this research. After having explained what their participation would consist of, the objectives of the study, voluntary nature, and confidentiality of the interview, participants signed an informed consent document to formally agree to participate and to be interviewed. The team of researchers with expertise in interviewing professionals, children, and young people, traveled to the different regions to conduct a face-to-face interview lasting between 40 and 60 min wherever it was most convenient for care leavers. The interviews were audio-recorded with the express consent of the participants.

The study has been performed in accordance with the ethical criteria of the Helsinki Declaration and the national legislation regarding personal data protection and was approved by the Research Ethical Committee of the University of Oviedo.

### 2.4. Data Analysis

Descriptive statistics for sociodemographic characteristics and bivariate analyses for differences between the UYM and CG were carried out using Chi-square for categorical variables and Student’s *t*-test for continuous variables. Cramer’s V and Cohen’s d were used to calculate effect size and the level of significance was established as *p* ≤ 0.05. The Statistical Package for Social Science IBM SPSS Statistics [29] was used to analyze data.

## 3. Results

### 3.1. Victimization and Child Care Background

According to the interview, the UYM group had significantly lower percentages in all types of maltreatment, with physical neglect the most common (28%), while emotional neglect and abuse, and physical abuse were the most common types for the CG. Significant differences were also detected for suffering multiple forms of maltreatment (UYM: 16.7%; CG: 74.2%) (Table 1).

As for their experiences in child care, the reason for admission was different for each group. In the case of the UYMs, admission was due exclusively to the fact that they were unaccompanied minors, while the causes were more diverse for young people in the CG, most of whom entered care due to neglect and/or abuse (81%), abandonment (8%), lack of parental control (8%), and filio-parental violence (3%). Significant differences were also revealed with respect to the time spent in out-of-home placement. UYMs entered child care at an older age (*M* = 15.90, *SD* = 1.65) than the CG (*M* = 10.17, *SD* = 5.29) [*t* (85.68) = −8.74., *p* ≤ 0.001]. In particular, 79% of UYMs entered at the ages of 16 or 17, while most of the CG (43%) were between zero and ten years and 38% were between 11 and 15 years of age. Another significant difference has to do with the duration of stay. Almost all the UYMs (91%) left child care within three years, after an average stay of 2 years (*M* = 2.10, *SD* = 1.65), whereas the CG had significantly longer stays (*M* = 7.83, *SD* = 5.29) [*t* (85.68) = 8.74., *p* ≤ 0.001].

### 3.2. Health and Risk Behaviors

Differences were significant in terms of participants’ current physical health status, as only one UYM had a serious health problem versus 22% of CG (Table 2). Chronic, physical illnesses, such as asthma, were the most common. Similarly, there were significant intergroup differences concerning intellectual disability as it was only present in the CG (8%). Very few UYMs had received any mental health treatment in the past (6%) and even fewer continued to receive it (4%). The CG were significantly more referred to treatment, both in the past (74%) and at the time of interview (33%). Suicide attempts emerged as an extremely serious problem and was reported by 7.7% in the CG and by 1.5% of the UYMs (statistically non-significant due to the relatively low frequencies), as well as suicidal ideation, that reached significant intergroup differences, with UYMs exhibiting a lower incidence (UYM: 3%; CG: 17%). Significant differences were likewise detected with respect to other risk behaviors: UYM reported less substance use (9%), with cannabis the most common, and they had fewer problems with the law (8%) than the CG (66%) for delinquent activity consisting of robberies or fights. Finally, the prevalence of unplanned pregnancy by a partner was fairly similar in both groups, without significant differences.

### 3.3. Situation of Young People in Their Transition to Adulthood

The current educational and occupational situation was similar across groups (Table 3), with continuing studies and training being the most frequent (UYM: 62%; CG: 45%). Approximately 16% of both groups were only working, and some were combining both studies and work (UYM: 9%; CG: 25%). Despite failing to reach statistical significance, it seems that UYM have more problems combining both activities. Finally, some 13% in both groups were neither studying nor working.

If we break the numbers down by type of studies or training, most UYMs attended some basic vocational training (65%), focused on gaining rapid employment either in the technical (mechanic, gardening, etc.) or hospitality field (restaurants, bars, etc.) and few had any form of intermediate vocational training or were finishing high school (25%). In contrast, the young people in the CG had more intermediate and advanced vocational training in several areas and 14% of them were studying at the university, which did not happen with any of the UYMs. Moreover, most of the CG (82.4%) wanted to continue studying, a percentage that was almost halved among UYMs (54%), with significant differences between groups (χ^2^ = 9.377, *p* = 0.002). However, more than one third (38.2%) thought that they would have serious obstacles to continue studies, given that they needed to work and earn money.

On the other hand, among those who stopped studying, basic vocational training was the most commonly achieved level among UYMs (47%; CG: 15%), while intermediate vocational training was the most common among young people in the CG (35%; UYM: 12%) and many of the young people in both groups had only obligatory or secondary studies (CG: 50%; UYM: 41%), with no significant differences between them. Furthermore, most of both groups, especially the CG (86%; UYM: 75%) wanted to resume their studies in the future, although the differences were not statistically significant. In any case, more than half of both groups thought it would be difficult, given their need to work and earn money.

With respect to work, significant differences were detected (χ^2^ = 4.104, *p* = 0.043) in the sense that employment rates were higher for the CG (41%) versus UYM (25%), regardless of whether they were only working or combining work and study. Employment in both groups was predominantly in the technical and hospitality fields, often with part-time (67%) or temporary contracts (64%), with a salary that did not reach EUR 500 for 40% of the participants, revealing no significant intergroup differences in these aspects. However, significant differences did emerge with respect to the jobs they aspired to attain in the future (χ^2^ = 17.414, *p* = 0.002). Both were oriented especially toward jobs in hospitality (UYM: 39%; CG: 32%) albeit there were also more UYMs who were pursuing technical employment (UYM: 28%; CG: 14%) or who stated that they had no preference (UYM: 23%; CG: 14%). Meanwhile, young people in the CG were more focused on jobs in health and socio-psychological fields (CG: 22%; UYM: 5%) or other categories (CG: 19%; UYM: 6%) such as security or computing.

Differences were also significant with respect to both the type and amount of income. UYMs more frequently received pocket money from their residential facility (60%; CG: 14%), while youth in the CG received aftercare financial assistance to a greater extent (47%; UYM: 12%). This was reflected in their income level in that the UYMs had less income each month. Consequently, significant differences were likewise detected in their ability to save money, with UYMs having less savings.

With respect to housing, the difference failed to reach statistical significance: both groups largely lived in apartments offered by aftercare agencies for care leavers, more so in the case of UYMs (56%; CG: 38%) or in extended care. Moreover, young people in the CG more often started living on their own in a rented apartment (26%; UYM 13%).

Regardless of these differences, most young people enjoyed stable placement after leaving care, as reflected by no changes (66%) or between one and two (23%) placement changes, while 11% had three or more changes, without significant differences between the two groups.

### 3.4. Social Support Network

Significant differences were found in many aspects related to the participants’ social support networks (Table 4). Concerning family, nearly all the UYMs had contact with their parents (95%) versus 69% of the CG, similar to their responses when asked about their siblings. Furthermore, most of the participants rated their relationship with their family as being positive, especially the UYMs. Nevertheless, little more than half considered their family to be a source of support, with no significant differences between groups. Regarding other sources of support, many care leavers (85%) mentioned friends, but a significantly larger proportion of youth in the CG stated that they could count on this kind of support. As for having an adult of reference to rely on in cases of need, the differences between groups were significant. UYMs primarily referred to social educators (aftercare or child care staff) as a reference figure (83%), while young people in the CG mentioned educators (45%), but also other figures, such as relatives (22%), acquaintances (21%), and in last place, their parents (12%). UYMs never mentioned their parents in this regard.

### 3.5. Aftercare Support

In our sample, care leavers spent a mean of 1.4 years (*SD* = 1.24) benefiting from the aftercare support. The percentage of young people who received such support for a prolonged period was low, especially among UYM (Table 5). Only 14% of UYM received some kind of aftercare benefit for two or more years, compared to 37% in the CG, with statistically significant differences. Participants benefited from one or multiple benefits offered by regional agencies, according to their demands and needs. Education and training guidance was the most common service used by care leavers in the sample, followed by support for integration into the labor market, the provision of accommodation, and legal assistance. Differences regarding legal and financial support were significant, highlighting the fact that UYMs received more legal assistance, while the young people in the CG accessed economic benefits more often. Moreover, those in the CG also received more psychological support, although the difference was not statistically significant.

## 4. Discussion

The UYM in our sample are males who arrived in our country, often close to majority age, predominantly from the Maghreb (especially Morocco) and Sub-Saharan countries, with demographic profiles similar to those found in other national studies [12,30]. As UYMs, they do not need to apply for asylum nor are they considered refugees, as in other countries, given that they are under the guardianship of the regional authorities and afforded the consideration and protection as any other unprotected child, in accordance with the national law of child protection. However, as soon as they come of age, they are no longer considered looked-after minors but adult migrants, and in order to stay and access the same resources as other young people they have to request the renewal or concession of the residence permit. In order to get this permit, they must meet different criteria depending on their condition (article 197, 198), such as having a positive report from the childcare agencies to certify their engagement and integration, having sufficient financial resources to support themselves during the validity of the permit, or having received an offer of employment contract during this time, etc. (Organic Law 4/2000). This implies that, as care leavers, their labor integration and access to aftercare services should be favored, but conversely, migration policies hinder their social insertion.

As for their personal history, the UYMs suffer fewer experiences of abuse and neglect than the CG, which, in contrast, display high rates of all the types of victimization experiences. A young boy from CG, for example, said, “*first I was living with my mother, who maltreated me, so they gave guardianship to my father, who neglected me. None of them have done well*”, similarly another said, “*I entered a center because my parents abused me, they had financial problems, my mother also had mental health problems after my father’s death, and they both had alcohol problems*”. This is consistent with the findings of Fernández-Artamendi et al. [31] regarding the high rates of victimization and polyvictimization of adolescents in residential child care. Therefore, although both UYMs and young people in CG are looked after, two completely different profiles can be observed, as also evidenced by Söderqvist [20]. The UYMs came into child care due to a migratory project to look for a job and future opportunities in a new country, with relatively few experiences of abuse and neglect, unlike the CG, who had to endure severe abuse and neglect in order to be in out-of-home care. The nature of UYMs’ immigration project and objectives is also reflected in their later admission and shorter stay in child care compared with other care leavers in our sample, as also shown by González-García et al. [26]. Residential care was practically the only resource for this specific group [12,20], due to the shortage of family foster placements in Spain, particularly for adolescents.

The UYMs in our sample also had a better health status, both physically and psychologically, as reflected in their lower rates of psychological treatment and lower incidence of suicidal behaviors with respect to their peers. Moreover, they presented lower rates of substance use and delinquency, confirming other authors’ conclusions that substance abuse and criminality are not substantial problems within this group [32]. These results are in line with those of Keles et al. [33] that point to the great resilience of UYMs, which enables them to do well despite the additional stressors that could expose them to mental health problems. However, other authors have detected high rates of psychological distress in unaccompanied adolescents [12]. Such differences in results may be due to several factors, such as having suffered fewer traumatic experiences [23] or the possibility that their mental health problems have abated after their arrival [34] and the participants’ different ages. Moreover, the UYMs with a good family functioning and relationship usually are better able to endure adversity [35]. It could also explain our results, since most of those who come to our country had a previous stable family situation, although with economic difficulties [36]. In this regard, an UYM said, “*We have a good relationship, my mother is very brave, she always wants to help us, I feel that she is suffering to help us and I want to help her*”. Be that as it may, the journey itself and the adaptation to the host country can cause sequelae [12], therefore, a careful exploration of their needs and psychological distress is needed, always keeping in mind the barriers that could hinder their understanding and how each culture handles emotions and psychological problems.

Concerning their integration, the UYMs in our sample have lower educational levels than their peers and are usually in a rush to start working, which is in keeping with the results of other studies about their preference for vocational training which facilitates a swift entry into the labor [26,37]. Nonetheless, there are fewer employed UYMs in our sample compared to their peers, which is reflected in their lower income levels. Along the same line as our results, other authors have found that migrant care leavers have worse results in these key areas compared to the rest of care leavers [38,39]. This is understandable considering their cultural background [20], the different opportunities for education in their country of origin [37], and the impact their administrative status has on their opportunities to access employment in the host country [40]. Their lower educational level left them ill-prepared for the competitive job market [41], which translates into lower employment rates and earnings [39,42,43,44], with the incumbent increased risk for negative outcomes [45], as for others care leavers. Moreover, their difficulties are compounded, since they need a residence and work permit in order to get a job, however in Spain, obtaining one does not necessarily imply obtaining the other. Hence, they may leave care without a work permit, but cannot get one without having a one-year, full-time contract, which is a challenge in and of itself considering the economic crisis and the care leavers personal barriers [37]. Some UYMs have mentioned that “*the complicated thing is the documentation, which takes a long time*”, or “*I can’t work because I don’t have the permit, and I don’t know if I will have it*”, or “*I don’t work, I’m looking for it but I need the one-year contract and it’s hard to find it*”. In the worst-case scenario, they leave care with an irregular legal situation, unable to work, having no place to live, and running the risk of being repatriated [19], all of which increases their vulnerability. These young people’s education and language skills must necessarily be improved to make a difference for their future insertion in the labor market and society in general. However, it should be noted that the UYMs in our sample tend to be younger than those in the CG, therefore some poor results could also be due to this age difference, since the older care leavers are, the more independent they become [46].

As for social support, the UYMs in our sample had more contact with their family and better relationships with them than their CG counterparts. These findings are consistent with what is known from the literature, as national care leavers usually have a complex relationship with their parents and receive limited or no support from them [47], while immigrant youth families continue to be an emotional reference for UYMs, despite the distance [18]. In this regard, for example, a young man from the CG said, “*there were many problems and uncomfortable situations, for which I have taken distance from my mother*”, while an UYM said, “*what gives me the strength to fight is my family, not with money or physically, but mentally*”. It is interesting to observe the clear difference between emotional support, which is maintained despite the distance for UYMs, and the lack of instrumental support due to the distance and their preference not to talk to the family about their problems, so as not to worry them. In this respect, we report the words of one care leaver who said, *“the truth is that I do not usually count on them, because it is useless to tell them [about] my things if I do not live with them, because they would feel bad too”.* On the other hand, UYMs’ social support network is based on educators and professionals, while young people in the CG have a more varied and peer-based support network. This limited social network of the UYMs in our sample may be the result of their short stay in the country, language barriers, or their reluctance to talk about their problems. Knowing the crucial role and protective function of social support for care leavers [48,49], improving informal support through mentoring relationships can be a beneficial option to assist both groups, but particularly UYMs, in coping with the multiple challenges they face in different life domains and expand their network [50].

As for the aftercare support received from care leavers in our sample, UYMs spent less time receiving such support. This can possibly be accounted for by their younger age than the CG in our study, although having found few UYMs older than 20 years benefiting from aftercare support can have a double explanation: first, that they become independent sooner or, conversely, that they disengage sooner from aftercare service because they are tired of having to obey rules. For example, one care leaver commented in this regard: *“I live on my own, since I left the center, I have lived where I could, with friends. They offered me to live in the apartment for care leavers, but I declined because I did not want any more rules”.*

Financial support was the support the UYMs in our sample benefited from the least, whereas legal advice was the most common, given their specific need for help obtaining a residence and work permits. The lower rate of financial support probably reflects the special requirements they must access for this type of help. Oftentimes, they do not meet certain criteria, for instance, having been in care for three years or more, being legal residents in the territory, and having a work plan, usually related to higher education and training, all elements that are often lacking in this group because of their immigrant status. It may be also due to differences between regions in the endowment of these programs, as found by another national study [12] or, in the worst case, there could be some degree of inequality in the support provided to these young people. Other studies similarly suggested that UYMs may have fewer chances to receive some form of aftercare support [25,40].

According to Spanish law, all care leavers must be supported during the transition process, both before and after leaving care. They must receive training and support for leaving care from 16 years old, and be supported after coming of age by means of different programs aimed to meet their needs in core domains. Based on these directives, the Autonomous Communities implemented programs to support care leavers in education, accommodation, social and labor insertion, economic income and psychological support. Nevertheless, local legal frameworks to regulate these measures were sometimes lacking. This translates into a disparity of criteria and available benefits between territories, which make it possible for young people to receive substantially or significantly lower support (e.g., financial) depending on the region in which they are located. Therefore, there is an awareness that preparation for independent living is crucial for their success in life [51], although in fact, they are not always properly supported [52]. Given the profile and well-defined objectives of this group, it is important to bolster the aftercare support services of each region and unify protocols in order to offer them a better and equal opportunity.

Although the findings presented in this paper are in line with what we expected in our hypothesis and with previous literature, some limitations must be acknowledged. Firstly, our results must be taken with caution because of the non-probabilistic sampling. Moreover, it must be remembered that young people who, like our participants, have access to aftercare services tend to be those who have the best chance of taking advantage of such opportunities and that they voluntarily agreed to participate in this study. These factors suggest that they may be among care leavers with a better profile and that a different picture could have been found by interviewing care leavers who suddenly disengaged after turning 18 and refused any help or follow-up support. The invisibility of this extremely vulnerable group is a common difficulty in research in this field. Furthermore, gender has not been taken into account, since not enough migrant females were found among regions, which reflects their scarce presence in child care, due to the masculinization of the migratory phenomenon.

## 5. Conclusions

Transition to adulthood from care is an issue that in recent decades has gained ground in international investigations. Nonetheless, there is still much to explore, especially when it comes to young unaccompanied migrants, which have not yet received the attention they deserve.

This paper contributes to awareness of the profiles, needs, and differences of unaccompanied migrant care leavers compared to Spanish natives or accompanied migrants. UYMs arriving in Spain have been found to come mostly from African countries (particularly Morocco), undertaking the migratory journey close to the majority age, aiming to improve their living conditions and achieve a more prosperous future than they could have in their native country. They have typically not had particularly traumatic experiences in their countries, or at the hands of their family, which is reflected in their exhibiting less psychological distress and treatments compared with the CG, who have suffered high rates of abuse and neglect and suffer more psychological distress. Their clear objective of obtaining permits and finding a job to take care of themselves and their families is reflected in a trajectory often free from risky behaviors, as well as in a shorter stay in care and aftercare compared to other care leavers. Nevertheless, UYMs had worse results than their peers in terms of education, which exposes them to lower employment rates and less income. They also appear to have a more limited support network in the host country, but a better relationship with their families. Findings with respect to the aftercare support received suggest that they may have more difficulties than their peers in accessing such support, especially certain types, probably because of their immigrant status. Currently, there is still a tension between protection and migration control policies [12]. On the one hand, the response to UYMs is framed in the UNCRC, pursuing without discrimination their best interests (article 2,3), and the enjoyment of all the rights included in the convention (article 22), however, such protection expires at the age of 18, at which immigration policies begin to prevail. Hence, this particularly vulnerable subgroup of care leavers can find themselves in a vicious circle of worse outcomes, having additional difficulties and stressors compared to their peers. Nevertheless, they also exhibit some strengths and high resilience, which may lead to think that, despite the difficulties, many of them may have a positive transition experience and fare quite well compared to other care leavers [23].

The results suggest the need to improve formal and informal supports to assist care leavers, and in particular, UYMs, in addressing the multiple challenges of transition. Improving and balancing the aftercare support services of different regions, to offer them better and equal opportunities, and adopt strategies to expand their support network should be priorities. Moreover, to promote their integration into the labor market, their educational level should be improved, instilling in them the importance of education and better qualification, as well as supporting them when they want to continue further studies and higher education. Furthermore, as UYMs are a dominant profile in the protection system, their special needs, difficulties, and cultures should be taken into account in the implementation of transition programs. Finally, to reap the fruits of previous work, it would be desirable to speed up and simplify the obtaining of permits in order to pursue their real best interest, facilitate their real integration in foster society, and avoid the risk of social exclusion.

Concerning further research, it might be interesting to carry out more studies focused on the process of transition and results obtained by those groups that are more invisible in care and aftercare, such as unaccompanied migrant girls and young people with a more complex profile, both nationals and migrants. It would also be interesting to use other types of instruments to assess constructs such as the well-being, psychosocial adjustment and life skills of this populations.

## Figures and Tables

**Table 1:** Victimization and Child Care Background. Differences between groups.

Variables	Total n (%)	CG n (%)	UYM n (%)	χ^2^	*p*	Effect Size Cramer’s *V*
**Maltreatment experienced** **^a^**						
Emotional neglect	64 (49.2)	54 (76.1)	10 (16.9)	45.04	<0.001	0.59
Emotional abuse	57 (43.8)	49 (72.1)	8 (12.9)	46.09	<0.001	0.60
Physical abuse	54 (42.2)	46 (69.7)	8 (12.9)	42.28	<0.001	0.58
Physical neglect	50 (41.0)	33 (53.2)	17 (28.3)	7.81	0.001	0.25
Exposure to gender violence	43 (36.1)	36 (57.1)	7 (12.5)	25.60	<0.001	0.46
Sexual abuse	19 (16.4)	16 (26.7)	3 (5.4)	9.60	0.002	0.29
Multiple forms	59 (46.8)	49 (74.2)	10 (16.7)	41.84	<0.001	0.58
**Age at entry**				54.59	<0.001	0.67
0–5 years	20 (14.4)	20 (27.8)	0 (0.0)			
6–10 years	13 (9.4)	11 (15.3)	2 (3.0)			
11–15 years	39 (28.1)	27 (37.5)	12 (17.9)			
16–17 years	67 (48.2)	14 (19.4)	53 (79.1)			
**Time in out-of-home placement**				58.08	<0.001	0.65
1–3 years	81 (58.3)	20 (27.8)	61 (91.0)			
4–6 years	22 (15.8)	18 (25.0)	4 (6.0)			
7–9 years	11 (7.9	10 (13.9)	1 (1.5)			
>9 years	25 (18.0)	24 (33.3)	1 (1.5)			
**Placement changes**				2.89	0.236	0.15
0	50 (36.5)	31 (42.5)	19 (29.7)			
1–2	53 (38.7)	24 (32.9)	29 (45.3)			
3 or more	34 (24.8)	18 (24.7)	16 (25.0)			

*Note*. CG = Comparison Group; UYM = Unaccompanied Young Migrants; χ^2^ = Chi-Square values; *p* = exact *p* values. ^a^ More than one category is possible.

**Table 2:** Health and Risk Behavior. Differences between groups.

Variables	Total n (%)	CG n (%)	UYM n (%)	χ^2^	*p*	Effect Size Cramer’s *V*
Physical health problems	17 (12.1)	16 (21.9)	1 (1.5)	13.89	<0.001	0.31
Intellectual disability	6 (4.3)	6 (8.2)	0 (0.0)	5.67	0.017	0.20
Current mental health treatment	27 (19.1)	24 (32.9)	3 (4.4)	18.43	<0.001	0.36
Past mental health treatment	58 (41.7)	54 (74.0)	4 (6.1)	65.75	<0.001	0.69
Suicidal ideation	13 (9.8)	11 (16.9)	2 (3.0)	7.22	0.007	0.23
Suicide attempt	6 (4.5)	5 (7.7)	1 (1.5)	2.92	0.112	0.15
Substance use	30 (21.3)	24 (32.9)	6 (8.8)	12.16	<0.001	0.29
Delinquent activity	29 (20.7)	24 (32.9)	5 (7.5)	13.74	<0.001	0.31
Unplanned pregnancy	8 (5.7)	5 (6.8)	3 (4.5)	0.37	0.546	0.05

*Note*. CG = Comparison Group; UYM = Unaccompanied Young Migrants; χ^2^ = Chi-Square values; *p* = exact *p* values.

**Table 3:** Situation of Care Leavers. Differences between groups.

Variables	Total n (%)	CG n (%)	UYM n (%)	χ^2^	*p*	Effect Size Cramer’s *V*
**Current situation**				7.01	0.072	0.22
Only studying	75 (53.2)	33 (45.2)	42 (61.8)			
Only working	23 (16.3)	12 (16.4)	11 (16.2)			
Working and studying	24 (17.0)	18 (24.7)	6 (8.8)			
Neither studying nor working	19 (13.5)	10 (13.7)	9 (13.2)			
**Studies and training**						
Current studies				21.75	<0.001	0.47
High School	11 (11.1)	5 (9.8)	6 (12.2)			
Intermediate/advanced vocational training	27 (27.3)	21 (41.2)	6 (12.5)			
Basic vocational training	49 (49.5)	17 (33.3)	32 (65.3)			
University	7 (7.1)	7 (13.7)	0 (0.0)			
Language	5 (5.1)	1 (2.0)	4 (8.3)			
Field				11.36	<0.001	0.52
Technical	33 (38.4)	9 (20.9)	24 (57.1)			
Hospitality	22 (25.9)	9 (20.9)	13 (31.0)			
Health and socio-psychological	10 (11.6)	10 (23.3)	0 (0.0)			
Others (sports, art, computers, etc.)	20 (23.3)	15 (34.9)	5 (11.9)			
**Work**						
Contract				2.656	0.265	0.24
Temporary	29 (64.4)	18 (62.1)	11 (68.8)			
Permanent	8 (17.8)	4 (13.8)	4 (25.0)			
Off-the-books	8 (17.8)	7 (24.1)	1 (6.3)			
Time				3.53	0.060	0.29
Part time	29 (67.4)	21 (77.8)	8 (50.0)			
Full-time	14 (32.6)	6 (22.2)	8 (50.0)			
**Income** (aside from salary)						
Typology						
Financial assistance	42 (30.0)	34 (46.6)	8 (11.9)	19.96	<0.001	0.38
Pocket money	50 (35.7)	10 (13.7)	40 (59.7)	32.20	<0.001	0.48
Other	9 (6.4)	6 (8.2)	3 (4.4)		0.355	
Amount				20.84	<0.001	0.45
Less than 300€	67 (65.7)	20 (42.6)	47 (85.5)			
From 300 to 700€	32 (31.4)	25 (53.2)	7 (12.7)			
More than 700€	3 (2.9)	2 (4.3)	1 (1.8)			
Savings	92 (65.2)	54 (74.0)	38 (55.9)	5.08	0.024	0.19
**Housing**						
Typology				5.50	0.139	0.19
Housing support	66 (46.8)	28 (38.4)	38 (55.9)			
Rent apartment	28 (19.9)	19 (26.0)	9 (13.2)			
Extended care	42 (29.8)	23 (31.5)	19 (27.9)			
Other	5 (3.5)	3 (4.1)	2 (2.9)			

*Note*. CG = Comparison Group; UYM = Unaccompanied Young Migrants; χ^2^ = Chi-Square values; *p* = exact *p* values.

**Table 4:** Support Network. Differences between groups.

Variables	Total n (%)	CG n (%)	UYM n (%)	χ^2^	*p*	Effect Size Cramer’s *V*
Contact with parents	110 (81.5)	50 (69.4)	60 (95.2)	14.82	<0.001	0.33
Contact with siblings	105 (78.4)	44 (62.0)	61 (96.8)	23.91	<0.001	0.42
**Quality of relationship**				12.59	<0.001	0.30
Positive	112 (83.0)	52 (72.2)	60 (95.2)			
Negative	23 (17.0)	20 (27.8)	3 (4.8)			
Support from family	76 (56.3)	44 (61.1)	32 (50.8)	1.45	0.228	0.10
Support from friends	116 (84.7)	68 (93.2)	48 (75.90)	8.66	0.003	0.25
Support from partner	38 (27.5)	20 (27.4)	18 (27.7)	0.002	0.969	0.00
**Reference adult**				17.78	<0.001	0.41
Educators	65 (61.9)	26 (44.8)	39 (83.0)			
Parents	7 (6.7)	7 (12.1)	0 (0.0)			
Other relatives	16 (15.2)	13 (22.4)	3 (6.4)			
Acquaintances	17 (16.2)	12 (20.7)	5 (10.6)			

*Note*. CG = Comparison Group; UYM = Unaccompanied Young Migrants; χ^2^ = Chi-Square values; *p* = exact *p* values.

**Table 5:** Aftercare Support. Differences between groups.

Variables	Total n (%)	CG n (%)	UYM n (%)	χ^2^	*p*	Effect Size Cramer’s *V*
Aftercare support				13.61	0.001	0.312
0–1 year	104 (74.3)	46 (63.0)	58 (86.6)			
2–3 years	26 (18.6)	22 (30.1)	4 (6.0)			
4 or more years	10 (7.1)	5 (6.8)	5 (7.5)			
Social education support	115 (84.6)	60 (83.3)	55 (85.9)	0.18	0.675	0.04
Labor integration	83 (61.5)	44 (62.0)	39 (60.9)	0.02	0.902	0.01
Accommodation	81 (57.4)	38 (52.1)	43 (63.2)	1.80	0.180	0.11
Legal assistance	71 (52.2)	17 (23.3)	54 (85.7)	52.82	<0.001	0.62
Financial help	49 (34.8)	37 (50.7)	12 (17.6)	16.95	<0.001	0.35
Psychological support	13 (9.2)	10 (13.7)	3 (4.4)	3.63	0.057	0.16

*Note.* CG = Comparison Group; UYM = Unaccompanied Young Migrants; χ^2^ = Chi-Square values; *p* = exact *p* values.

## Data Availability

The data presented in this study are available from the corresponding author upon reasonable request, due to privacy and ethical reasons.
